# The Endoplasmic Reticulum Membrane Protein Complex Is Important for Deoxynivalenol Production and the Virulence of *Fusarium graminearum*

**DOI:** 10.3390/jof11020108

**Published:** 2025-01-31

**Authors:** Lei Chen, Yaxian Liu, Yu Wang, Yaxin Zhang, Saisai Wang, Liyuan Zhang, Kai Lu, Xiaochen Chen, Hansong Dong, Shenshen Zou

**Affiliations:** 1 Department of Plant Pathology, College of Plant Protection, Shandong Agricultural University, Tai’an 271018, China; chenlei@sdau.edu.cn (L.C.); 2023110107@sdau.edu.cn (Y.L.); 2022110112@sdau.edu.cn (Y.W.); 2022110119@sdau.edu.cn (Y.Z.); 2021120154@sdau.edu.cn (S.W.); lyzhang@sdau.edu.cn (L.Z.); lukai@sdau.edu.cn (K.L.); chenxc66@sdau.edu.cn (X.C.); hsdong@sdau.edu.cn (H.D.); 2 National Key Laboratory of Wheat Improvement, Shandong Agricultural University, Tai’an 271018, China

**Keywords:** *Fusarium graminearum*, mycotoxin, EMC, virulence, doxynivalenol (DON)

## Abstract

*Fusarium graminearum* is recognized as the pathogen responsible for wheat head blight. It produces deoxynivalenol (DON) during infection, which endangers human health. DON biosynthesis occurs within toxisomes in the endoplasmic reticulum (ER). In eukaryotes, the ER membrane protein complex (EMC) is critical for the ER’s normal operation. However, the specific role of the EMC in *F. graminearum* remains poorly understood. In this study, six EMC subunits (FgEmc1-6) were identified in *F. graminearum*, and all of them were localized to the toxisomes. Our results demonstrate that the EMC is indispensable for vegetative growth and asexual and sexual reproduction, which are the fundamental life processes of *F. graminearum*. Importantly, EMC deletion led to reduced virulence in wheat spikes and petioles. Further investigation revealed that in Δ*Fgemc1-6*, the expression of trichothecene (*TRI*) genes is decreased, the biosynthesis of lipid droplets (LDs) is diminished, toxisome formation is impaired, and DON production is reduced. Additionally, defects in the formation of the infection cushion were observed in Δ*Fgemc1-6*. In conclusion, the EMC is involved in regulating growth and virulence in *F. graminearum*. This study enhances our understanding of the EMC functions in *F. graminearum* and offers valuable insights into potential targets for managing wheat head blight.

## 1. Introduction

*Fusarium graminearum* is widely recognized as the principal pathogen accountable for wheat head blight. Besides diminishing wheat yield, this pathogen also produces a variety of mycotoxins during the process of infecting the host [1,2,3]. Deoxynivalenol (DON) has been extensively investigated among these toxins and is regarded as the most detrimental contaminant [4,5]. DON inhibits protein biosynthesis, and even small levels of DON can trigger acute symptoms such as nausea, vomiting, and diarrhea, while excessive intake can lead to acute toxicity and may endanger the lives of humans and mammals [6,7]. Furthermore, DON is a crucial pathogenic factor of *F. graminearum*, contributing significantly to the spread of this pathogen within the wheat spike during infection at the flowering stage [8]. Therefore, the identification of regulatory mechanisms of DON biosynthesis in *F. graminearum* may provide novel and effective strategies in the management of wheat head blight and mycotoxin contamination.

The endoplasmic reticulum (ER), as the largest organelle in eukaryotic organisms, serves as the primary site for the biosynthesis of numerous secondary metabolites. In *F. raminearum*, the biosynthesis of DON is intricately associated with the ER [9]. When *F. graminearum* initiates the plant infection, the ER undergoes a remodeling process, giving rise to specialized structures known as toxisomes, which are reportedly the sites where DON is biosynthesized [9,10,11]. Proteins that regulate ER function and structure are crucial for toxisome formation and impact DON biosynthesis, such as the ER fusion protein FgSey1 [12], the ER lipid droplet formation-associated proteins FgPah1 and FgNem1 [13], and the ER-located FgHmr1, which encodes a hydroxymethylglutaryl (HMG) CoA reductase involved in the mevalonate pathway [14]. Furthermore, essential regulatory proteins like Tri1 and Tri4 in the DON biosynthesis pathway are also located in toxisomes, facilitating DON production. Despite the significant role of the ER in DON biosynthesis, the specific regulatory mechanisms by which ER-associated proteins influence toxisome formation remain largely unexplored.

The ER membrane protein complex (EMC) consists of a set of highly conserved subunits located in the ER [15]. As an insertion enzyme or chaperone for membrane proteins, the EMC is crucial for sustaining lipid equilibrium, promoting signal transduction, affecting disease progression, and bolstering protein biosynthesis [16,17,18]. While the roles of the EMC have been extensively documented, it is still unknown whether the EMC contributes to the regulation of toxisome formation and DON production in *F. graminearum*. Furthermore, the impact of EMC on the pathogenicity of *F. graminearum* is not well understood.

In our study, we identified the existence of six EMC subunits located on the toxisomes in *F. graminearum*. Our findings demonstrate that the EMC is essential for various biological processes in *F. graminearum*, such as vegetative growth, asexual and sexual reproduction, and virulence. The deletion of *FgEMC1-6* led to a reduction in DON content, a decrease in toxisome formation, and a diminished generation of lipid droplets (LDs) integral to toxisome formation. Furthermore, the infection structures of the pathogen in the Δ*Fgemc1-6* mutants exhibit abnormalities. Therefore, our data suggest that the EMC plays a significant role in the growth and virulence of *F. graminearum*.

## 2. Materials and Methods

### 2.1. Strain and Culture Conditions

The PH-1 was used in this experiment as a wild-type strain [19]. The EMC mutant strains and PH-1 of *F. graminearum* were incubated on potato dextrose agar (PDA) media at a temperature of 25 °C in a light-free environment. The colony diameter of V8 medium, complete medium (CM), 5 × yeast extract–glucose (YEG), and minimal medium (MM) colonies was determined after 3 days at 25 °C, and the height of the aerial hyphae within a test tube containing PDA was measured after 3 days at the same temperature. Stress evaluations were conducted by observing the growth under diverse stress conditions on CM supplemented with KCl, Congo Red (CR), hydrogen peroxide (H_2_O_2_), sorbitol, and dithiothreitol (DTT). In the pigment observation experiment, the fungus cake was incubated in a PDB culture medium for three days to monitor color alterations [20]. All the experiments were replicated thrice.

### 2.2. Generation of EMC Mutants

The split-marker approach was employed to generate EMC deletion mutants. Specifically, 1000 bp upstream and 1000 bp downstream flanking sequences of *FgEMC1-6* were amplified by PCR. The hygromycin phosphotransferase (*HPH*) gene was also amplified from pCB1003. The primers used for PCR amplification are listed in Table S1. Subsequently, overlapping PCR was utilized to construct the *FgEMC1-6* gene replacement constructs. Then, the PCR products were transformed into protoplasts of the wild-type (WT) PH-1 through polyethylene glycol (PEG)-mediated transformation [21]. The resulting transformants were screened on a TB_3_ medium supplemented with hygromycin. Eventually, Δ*Fgemc1-6* was validated using the specific primers presented in Table S1.

### 2.3. Phylogenetic Evolutionary Tree Analysis

We retrieved protein sequences from ten species including *F. graminearum*, F. oxysporum, Saccharomyces cerevisiae, Homo sapiens, Arabidopsis thaliana, Neurospora crassa, Aspergillus nidulans, Magnapothe oryzae, Schizosaccharomyces pombe and Ustilago maydis on the NCBI website (https://www.ncbi.nlm.nih.gov/ accessed on 20 November 2024). Using the ClustalW program, we assessed the homology of EMC subunits among different species and constructed a phylogenetic tree with the MEGA11 software.

### 2.4. Assays for Asexual and Sexual Reproduction

For the purpose of obtaining spore counting statistics and conducting morphological observations, five fungus cakes were carefully transferred from fresh plates into carboxymethyl cellulose (CMC) liquid media and then incubated at 25 °C for a duration of 5 days. The spores were counted with a specialized counter after being stained with calcofluor white (CFW) for one minute, and the examination was performed using a fluorescence microscope [12]. This entire procedure was repeated three times to ensure reliability. In the experiments concerning the induction of sexual reproduction, the strain was inoculated onto carrot media (CA). Once the mycelium completely covered the plate, 500 μL of Tween 20 was added to the plate. Then, the mycelium was pressed onto the culture medium, ensuring it adhered as closely as possible. After this step, the plate was placed under a black light lamp to induce the formation of perithecia [22]. Subsequently, the morphology of the perithecia and ascospores was analyzed using a microscope [23]. This process was replicated three times to enhance the accuracy of the results.

### 2.5. Plant Infection Assays

Both the PH-1 and Δ*Fgemc1-6* strains were inoculated into CMC and cultured for 7 days. The conidia harvested from the culture were filtered, and deionized distilled water (ddH_2_O) was added to prepare a conidial suspension with a concentration of 2 × 10^5^ conidia ml^−1^. A volume of ten microliters of fresh spore suspension was inoculated onto the stigma of the wheat variety Jimai 22 during its flowering stage in the field. Images were taken two weeks post-inoculation, and the disease index was calculated by counting the number of diseased spikelets exhibiting symptoms of withering and white discoloration. Three days after inoculation, the cells were fixed using a 4% (*v*/*v*) glutaraldehyde and 2% (*v*/*v*) paraformaldehyde solution, followed by dehydration with varying concentrations of anhydrous ethanol. The samples were then sealed with isoamyl acetate, dried, and subjected to ion spraying. Subsequently, the sections were examined using a Japanese electron scanning electron microscope (JEOL, Tokyo, Japan) [24]. The wheat coleoptile experiment was conducted following previously established protocols [25]. In the wheat infection experiment, 30 wheat spikes or coleoptiles were inoculated.

### 2.6. DON Production Assays

In order to ascertain DON production, five mycelial plugs of fresh mycelia from Δ*Fgemc1-6* and PH-1 were independently inoculated into 5 g of sterile wheat grains and incubated at 25 °C over a period of 20 days. Subsequently, DON was extracted by the high-performance liquid chromatography (HPLC) method detailed in previous studies [12,26]. We determined the levels of DON and fungal ergosterol in each sample, with ergosterol content used as an internal control for the relative quantification of DON [27,28]. This experiment was conducted three times.

### 2.7. Gene Expression Analysis

PH-1 and Δ*Fgemc1-6* were cultivated in TBI for a duration of three days. The mycelium was then harvested for RNA extraction using an RNA-easy isolation reagent (R701, Vazyme, Nanjing, China), and 1 μg of each RNA sample was used for reverse transcription with a HiScript II 1st strand cDNA synthesis kit (R212, Vazyme, Nanjing, China) [20]. The expression levels of pigment biosynthetic genes (*AURJ*, *AURF*, *GIP1*, *GIP2*, and *PKS12*), as well as DON biosynthesis-related genes (*TRI1*, *TRI4*, *TRI5*, *TRI6*, and *TRI10*), were examined using RT-qPCR. *GAPDH* was employed as an internal control for expression levels. The specific primers used are as described previously [28] (Table S1). By applying the 2^−ΔΔCt^ method, the relative expression level of the target gene was calculated in accordance with the guidelines of the ChamQ Universal SYBR qPCR Master Mix (Q711, Vazyme, Nanjing, China).

### 2.8. Microscopic Observation

To investigate the effect of FgEmc1-6 on toxisome formation, the plasmid pYF11-FgTri1-GFP was introduced into PH-1 and Δ*Fgemc1-6* via PEG-mediated protoplast transformation, resulting in the generation of strains PH-1/FgTri1-GFP and Δ*Fgemc1-6*/FgTri1-GFP, both expressing the FgTri1-GFP fluorescent protein. To assess the co-localization of FgEmc1-6 with FgTri1-GFP, the pHD64-FgEmc1-6-mCherry plasmid was constructed and co-introduced with pYF11-FgTri1-GFP into PH-1 using the same transformation method. The primers used for vector construction are listed in Table S1. The strains were cultured in CM or TBI medium for 24 h, after which fluorescence signals were observed using fluorescence microscopy (Nikon, Japan) with GFP/RFP filters and an X40 object lens. For lipid droplet observation, PH-1 and Δ*Fgemc1-6* were incubated in CM and TBI medium for 48 h. Mycelia were subsequently harvested, transferred to Nile red dye, and incubated at 37 °C for 10 min, and lipid droplet production was assessed using the same microscopy with an X40 object lens and GFP filters.

### 2.9. Statistical Analyses

To ensure the reproducibility of the trends and relationships identified among the cultures, all experimental data were obtained from three independent samples. Each error bar represents the standard deviation (SD) calculated from the mean of triplicate samples. Statistical significance was assessed using Duncan’s multiple range test, with a significance level set at *p* < 0.05. The analysis was conducted using SPSS Statistics 26.

## 3. Results

### 3.1. Identification of F. graminearum EMC Subunits

In *F. graminearum*, six EMC subunits (FGSG_00261, FGSG_07429, FGSG_05601, FGSG_09736, FGSG_11940, and FGSG_01360) were identified using the full-length amino acid sequences of EMC in *S. cerevisiae* and *Homo sapiens* as queries by NCBI BLAST and were designated as FgEmc1-6, respectively (Figure S1). We further performed a protein domain analysis on the amino acid sequences of FgEmc1-6 using the SMART database (https://smart.embl.de/ accessed on 21 March 2020). The analysis demonstrated that FgEmc1 harbors one transmembrane domain (TMD), while FgEmc3, FgEmc4, FgEmc5, and FgEmc6 each possess two TMDs, aligning with the role of the EMC as either insertion enzymes or chaperones [29,30]. Furthermore, FgEmc1 features a pyrroloquinoline quinone (PQQ)-like repeat, potentially serving as a crucial scaffold for protein–protein interactions [31]. FgEmc2 has a tandem tetratricopeptide repeat (TPR) motif, potentially implicating its role in interacting with other proteins. Additionally, FgEmc5 contains a membrane magnesium transporter (MMgT) domain, which might modulate the physiological and adaptive responses of *F. graminearum* to magnesium [32]. Moreover, FgEmc1, FgEmc3, and FgEmc4 are anticipated to have uncharacterized function domains (DUF1620, DUF106, and DUF1077).

**Figure 1:**
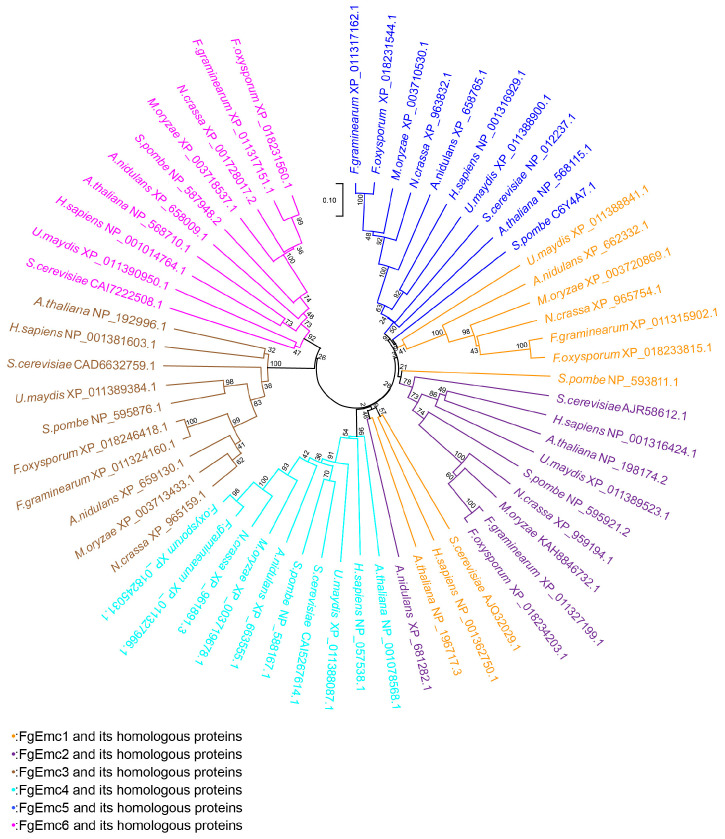
Phylogenetic analysis of the EMC subunits. A phylogenetic analysis of EMC subunits from diverse species was performed. The ClustalW program was used to compare the subunit sequences, and a phylogenetic tree was constructed with MEGA11 software. Sequences were obtained from *F. graminearum*, *F. oxysporum*, *Saccharomyces cerevisiae*, *Homo sapiens*, *Arabidopsis thaliana*, *Neurospora crassa*, *Aspergillus nidulans*, *Magnapothe oryzae*, *Schizosaccharomyces pombe,* and *Ustilago maydis*, facilitating a comprehensive exploration of the evolutionary relationships among EMC subunits. The scale bar = 0.10 indicates 10 differences per 100 amino acids.

The protein sequences of the EMC protein family in *F. graminearum* were compared with those from nine other species, and an evolutionary tree was constructed using MEGA11 software (Figure 1). The results showed that the EMC of *F. graminearum* had a relatively close phylogenetic relationship with other filamentous fungi besides *F. oxysporum* but a more distant one with that of *Saccharomyces cerevisiae*, *Homo sapiens,* and *Arabidopsis thaliana*, indicating the adaptive divergence and functional evolution of EMC protein families among different biological groups in evolution.

### 3.2. The EMC Is Involved in the Vegetative Growth of F. graminearum

In order to explore whether the EMC subunits affect the vegetative growth of *F. graminearum*, we created deletion mutants corresponding to each subunit within the EMC, which were then verified by PCR (Figure S2A,B).

**Figure 2:**
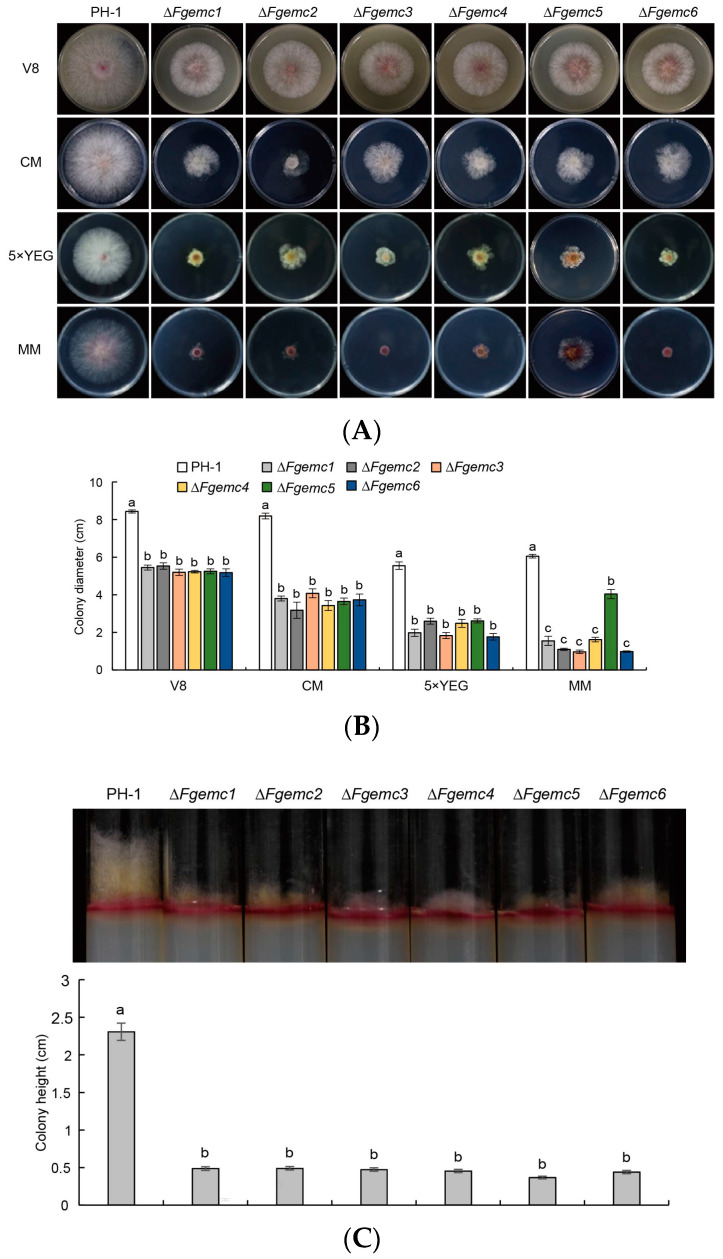
The EMC is involved in the vegetative growth of *F. graminearum*. (**A**) Colonies of PH-1 and Δ*Fgemc1-6* were cultured on V8, 5×YEG, CM, and MM plates at 25 °C for 3 days. (**B**) The colony diameters of each strain from (**A**) were quantified. (**C**) Aerial hyphae of the strains were grown in test tubes on PDA medium at 25 °C for 3 days, and the aerial hyphae of the indicator strains were quantified. For (**B**,**C**), SPSS Statistics 26 was used. Error bars represent the mean ± SD of three replicates. Lowercase letter differences denote statistically significant differences (*p* < 0.05).

A subsequent evaluation of the vegetative growth characteristics of these mutants demonstrated that Δ*Fgemc1-6* all displayed a diminished colony diameter on all four types of media in comparison to PH-1 (Figure 2A,B). In assessing the height of the aerial mycelium, a significant decrease was observed. The height of the aerial hyphae in PH-1 was measured at 2.306 cm, whereas the heights of the aerial hyphae in Δ*Fgemc1-6* were recorded as 0.486 cm, 0.490 cm, 0.473 cm, 0.453 cm, 0.366 cm, and 0.440 cm, respectively (Figure 2C). These findings imply that the EMC subunits play a crucial part in the vegetative growth process of *F. graminearum*.

### 3.3. The EMC Is Essential in the Response of F. graminearum to Diverse Environmental Stressors

**Figure 3:**
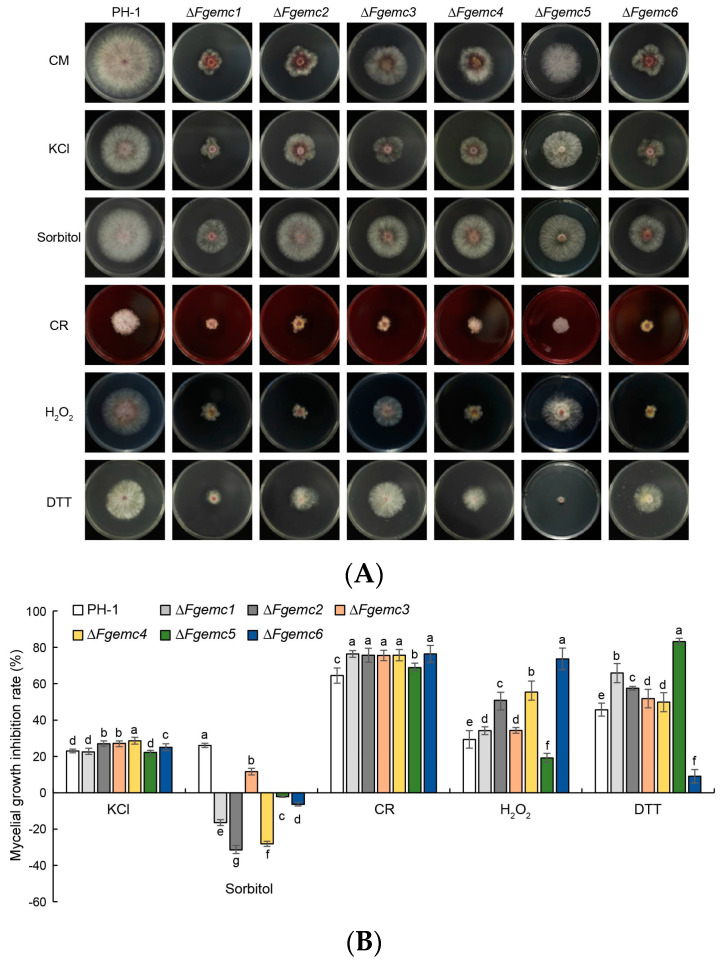
Vegetative growth of PH-1 and Δ*Fgemc1-6* in response to different stress conditions. (**A**) The indicated strains were inoculated on CM supplemented with KCl, sorbitol, CR, H_2_O_2_, or DTT. For each treatment, three plates were prepared, and the experiment was repeated three times. (**B**) Statistical analysis of mycelial growth inhibition rate of each treatment using SPSS Statistics 26 based on colony diameters in (**A**). The mycelial growth inhibition rate = [(colony diameter of PH-1 − colony diameter of Δ*Fgemc1-6*)/colony diameter of PH-1 × 100%]. The columns represent means, while the error bars indicate standard deviations (SDs). Lowercase letter differences denote significant differences (*p* < 0.05).

*F. graminearum* exhibits significant vulnerability to environmental stressors during its growth. To investigate the stress response of the EMC under various external conditions, the PH-1 strain and Δ*Fgemc1-6* mutants were inoculated into media containing several stress-inducing agents, including osmotic stressors (KCl and sorbitol), cell wall stressors (Congo Red, CR), oxidative stress (hydrogen peroxide, H_2_O_2_), and endoplasmic reticulum (ER) stress (dithiothreitol, DTT) (Figure 3A,B). Among the six mutants, Δ*Fgemc2-4* and Δ*Fgemc6* demonstrated increased sensitivity to KCl. Conversely, all six mutants exhibited reduced sensitivity to sorbitol. These findings indicate that the EMC plays a role in modulating the osmotic stress response of *F. graminearum*. Following treatment with the cell wall inhibitor CR, all six mutants displayed heightened sensitivity, suggesting that the EMC is also responsive to cell wall stress. Under H_2_O_2_ treatment, the growth inhibition of Δ*Fgemc1-4* and Δ*Fgemc6* strains was greater compared to PH-1, while the mycelial growth inhibition rate of Δ*Fgemc5* was diminished. Given that the EMC is classified as a group of ER membrane-like proteins, the sensitivity of the EMC mutants to ER stress was further examined by evaluating their responses to DTT. The results indicated that Δ*Fgemc1-5* was more sensitive to DTT, whereas Δ*Fgemc6* exhibited reduced sensitivity. Collectively, these results suggest that the EMC is involved in the response to osmotic stress, cell wall stress, oxidative stress, and ER stress.

### 3.4. The EMC Is Critical for the Generation of Conidia, Perithecia, and Ascospores in F. graminearum

**Figure 4:**
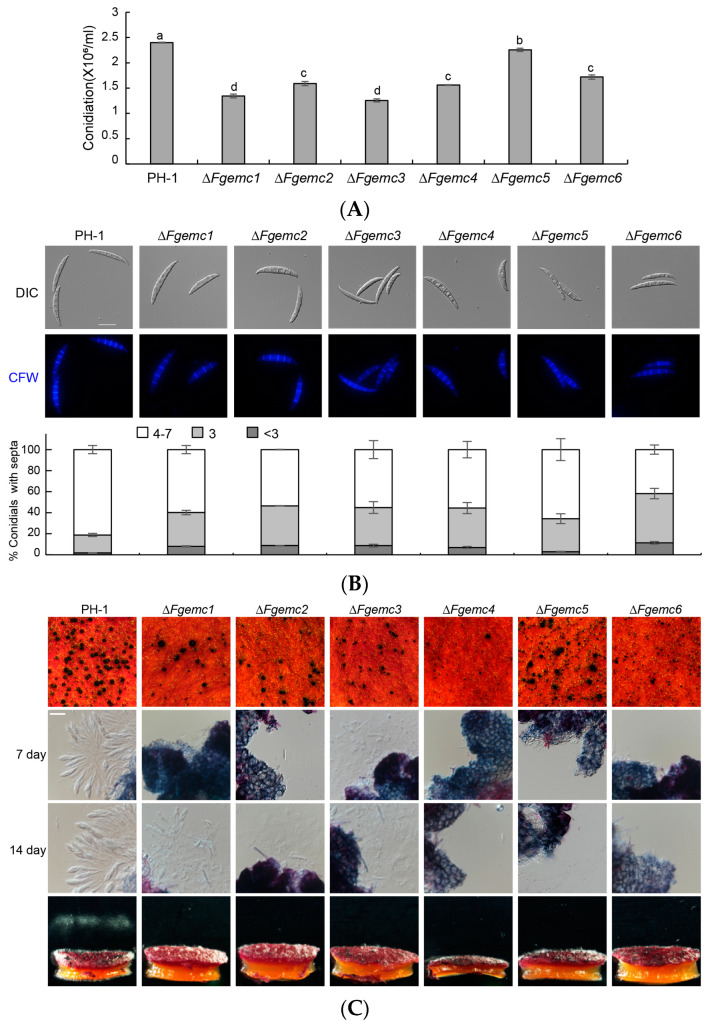
The EMC is required for asexual and sexual reproduction of *F. graminearum*. (**A**) Statistical analysis of conidia production by PH-1 and Δ*Fgemc1-6* in CMC liquid cultures at 25 °C for 5 days. The experiment was repeated three times. (**B**) Conidial morphology of the strains cultured in CMC liquid media at 25 °C for 5 days was observed. Fresh conidia were stained with CFW to visualize septa and examined using live-cell fluorescence microscopy. For each treatment, the number of septa in 200 conidia was counted. Statistical results are presented as the percentage of spores with different septa numbers: ≤2, 3, and 4–7. Columns show means, and error bars represent SDs. Scale bars are 20 μm. (**C**) Perithecia and ascospores of the strains were grown on carrot agar plates for 2 weeks, and ascospore discharge of the perithecia was examined. Scale bars are 20 μm. Lowercase letter differencesindicate significant differences (*p* < 0.05).

To further explore the role of the EMC in asexual reproduction, a comprehensive analysis was carried out on the production and morphology of conidia in both the PH-1 strain and the Δ*Fgemc1-6* mutant strains. The outcomes of this analysis indicated that there were significant reductions in conidia production in the Δ*Fgemc1*-*6* strains, with decreases of 44.1%, 36.1%, 45.8%, 35.4%, 25.8%, and 30%, respectively, when compared to the PH-1 strain (Figure 4A). The morphology of conidia was further investigated through CFW staining. Microscopic examination revealed that the PH-1 strain produced 81.2% normal conidia, characterized by the presence of 4-7 septa, while 18.8% of the conidia were abnormal, containing three or fewer septa. In contrast, the proportion of abnormal conidia was significantly higher in the Δ*Fgemc1-6* strains, with 40.1%, 46.5%, 44.9%, 44.4%, 39.5%, and 58.2%, respectively (Figure 4B). These results strongly suggest that the EMC is actively involved in the asexual reproduction process of *F. graminearum*.

Sexual reproduction in *F. graminearum* is an important component in the disease cycle of wheat head blight [33]. The PH-1 and Δ*Fgemc1*-*6* strains were cultured on a CA medium. Following full growth on the plates, the mycelium was flattened and induced with black light for 7 days, resulting in the production of perithecia in all strains, although the ΔFg*emc1*-*6* strains exhibited significantly fewer perithecia compared to PH-1. After 7 days, with the exception of Δ*Fgemc3*, which produced abnormal spores, the other five mutants did not generate any ascospores. After 14 days, ΔFg*emc1-3* failed to produce normal mature ascospores, while ΔFg*emc4*-*6* showed no ascospore production. Additionally, a test was conducted to assess the release of ascospores, which revealed that the abnormal spores produced by the Δ*Fgemc1*-*3* strains were unable to be released (Figure 4C). These findings suggest that the EMC plays a crucial role in the sexual reproduction of *F. graminearum*.

### 3.5. The EMC Plays a Crucial Role in Regulating F. graminearum Virulence

**Figure 5:**
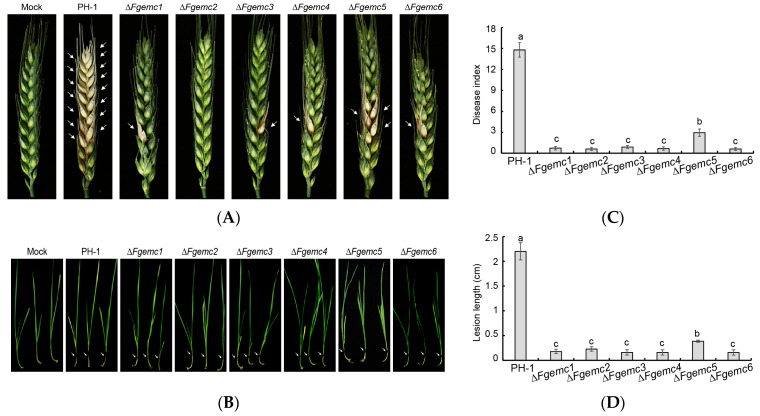
The EMC is required for full virulence of *F. graminearum*. (**A**) The virulence of PH-1 and Δ*Fgemc1-6* was determined by inoculating onto flowering wheat heads. The number of diseased spikelets (withered and white spikelets symptoms) per spike was measured 14 days after conidia inoculation. At least 30 spikelets were inoculated per strain. (**B**) Wheat coleoptiles were inoculated with conidial suspensions of the strains, and lesion lengths of the black areas at the base of the wheat were examined at 14 dpi. At least 30 wheat coleoptiles were inoculated per strain. Arrows were added to indicate the diseased parts in (**A**,**B**). (**C**,**D**) Statistical analyses of disease index on wheat spikes and lesion lengths on wheat germ sheaths of the corresponding strains in (**A**,**B**). Columns show means, and error bars represent SDs. Lowercase letter differences indicate significant differences (*p* < 0.05).

*F. graminearum* is identified as the primary causal agent of wheat head blight [34]. In order to determine whether the EMC is involved in the virulence of *F. graminearum*, we inoculated each strain into flowering spikes of the wheat variety Jimai 22, which was at the anthesis stage and growing in the field. Wheat spikes were inoculated with sterile water (Mock), a conidial suspension of the PH-1 strain, or a mutant strain. After 14 days of incubation, wheat spikes inoculated with PH-1 exhibited obvious lesions, while those inoculated with Δ*Fgemc1-6* strains only showed limited lesions at the inoculation site without spreading to other wheat grains through the rachis (Figure 5A,B). Additionally, a noticeable decrease in lesion length was observed in wheat germinal sheaths infected with the mutant strains (Figure 5C,D). These findings indicate that EMC plays an important role in the virulence of *F. graminearum*.

Furthermore, scanning electron microscopy examination of infection structures on the palea surface revealed that PH-1 formed an infection cushion (IC), while Δ*Fgemc1*, Δ*Fgemc3*, and Δ*Fgemc5* also formed ICs, albeit smaller in size compared to PH-1. On the other hand, Δ*Fgemc2* exhibited a lobate appressorium (LA), and Δ*Fgemc4* and Δ*Fgemc6* only had a foot structure (FS) without forming an IC (Figure S3). These results suggest that EMC influences the formation of infection structures, thereby impacting the infection process of *F. graminearum*.

### 3.6. The EMC Is Required for DON Production and Pigmentation in F. graminearum

**Figure 6:**
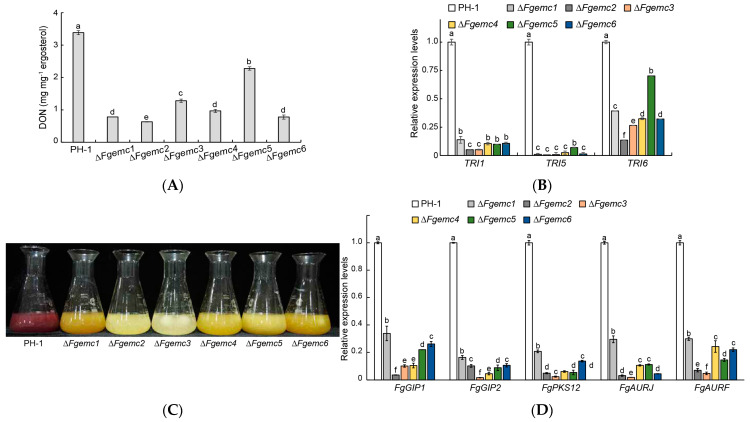
The EMC is important for DON biosynthesis and pigmentation of *F. graminearum*. (**A**) DON biosynthesis in wheat kernels was determined 20 days after infection with PH-1 and Δ*Fgemc1-6*. Ergosterol content served as an internal control. (**B**) The relative expression levels of *TRI1*, *TRI5*, and *TRI6* in PH-1 and mutants were determined by real-time qPCR. (**C**) Pigmentation of PH-1 and Δ*Fgemc1-6* was determined after 3 days of incubation in PDB media. (**D**) The relative expression levels of *FgGIP1*, *FgGIP2*, *FgPKS12*, *FgAURJ*, and *FgAURF* in the strains were analyzed. For A, B, and D, three different biological replicate samples were used. Columns show means, and error bars represent SDs. Lowercase letter differencesindicate significant differences (*p* < 0.05).

The significance of DON as a pathogenic factor in *F. graminearum* infection has been well documented in previous studies [35,36,37]. To investigate the potential relationship between the decreased virulence of the EMC mutants and DON biosynthesis, we examined the levels of DON in the EMC mutants compared to the PH-1 strain. Our results showed a significant decrease in DON generation in Δ*Fgemc1-6*, with reductions of 76.9%, 81.4%, 62.2%, 71.5%, 32.8%, and 76.9%, respectively (Figure 6A). It is known that the expression of *TRI* genes plays a crucial role in regulating DON biosynthesis [38]. Consequently, we proceeded with a detailed examination of the expression patterns of *TRI1*, *TRI5*, and *TRI6* genes. Our analysis revealed a marked reduction in the expression levels of these genes in Δ*Fgemc1-6* compared to the PH-1 strain (Figure 6B). These findings indicate that the EMC is implicated in regulating DON biosynthesis.

DON and pigments are fungal secondary metabolites [12]. After incubating the PH-1 and Δ*Fgemc1-6* strains in PDB for three days, a noticeable yellow coloration was observed in the incubation solution of the mutants, along with the downregulation of pigment biosynthetic genes (*FgGIP1*, *FgGIP2*, *FgPKS12*, *FgAURJ*, and *FgAURF*) as demonstrated in the mutants through RT-qPCR (Figure 6C,D). This indicates that the EMC plays a role in regulating the biosynthesis of secondary metabolites, such as DON and pigments, in *F. graminearum*, which is consistent with the observation that the absence of the EMC subunit reduces the virulence of *F. graminearum*.

### 3.7. The EMC Is Involved in the Toxisome Formation of F. graminearum

**Figure 7:**
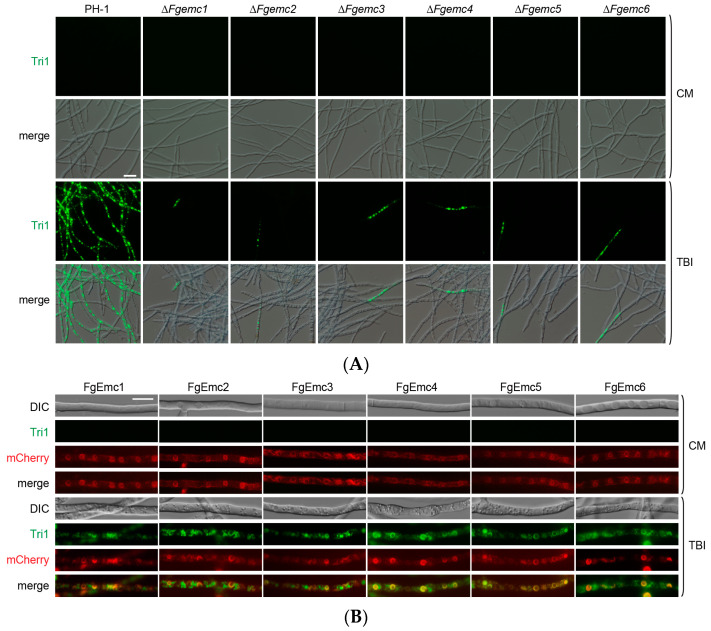
The EMC plays an essential role in the toxisome formation of *F. graminearum*. (**A**) Toxisome formation in PH-1 and Δ*Fgemc1-6* was determined. Vegetative hyphae of strains expressing Tri1-GFP were cultured in TBI or CM, and Tri1 localization was visualized by fluorescence. Scale bars are 10 μm. (**B**) Co-expression of Tri1-GFP with different FgEmc-mCherry strains in vegetative hyphae was studied. The strains were grown in CM or TBI media, and the colocalization of Tri1 and EMC subunits was determined by live-cell fluorescence microscopy. Scale bars are 10 μm.

In the mycotoxin biosynthesis of *F. graminearum*, Tri1 localizes to toxisomes, where it regulates DON production and serves as a marker [9,39]. We detected the localization of Tri1-GFP in each strain. In control conditions (CM), Tri1-GFP was not detected in either PH-1 or Δ*Fgemc1-6*. Upon TBI induction, we observed a significant increase in spherical and crescent-shaped Tri1-GFP structures in PH-1, while the number of these structures in Δ*Fgemc1-6* decreased notably compared to PH-1 (Figure 7A), suggesting a role for EMC in toxisome formation. Subsequently, we observed co-localization of FgEmc1-6 and Tri1-GFP. FgEmc1-6 displayed a typical ER structure in CM and co-localization with Tri1-GFP following TBI (Figure 7B).

Given that LDs are generated during toxisome formation [13], we examined whether mutations in EMC affect toxisome formation through LD biosynthesis. After TBI, PH-1 showed numerous LD signals, while the mutant exhibited significantly fewer signals (Figure S4), indicating that EMC regulates LD generation. Overall, these findings suggest that EMC plays a crucial role in both toxisome formation and LD generation, which is consistent with its involvement in DON biosynthesis.

## 4. Discussion

*F. graminearum* produces a range of mycotoxins, with deoxynivalenol (DON) identified as the most detrimental. This mycotoxin serves not only as a significant pathogenic factor but also poses a considerable risk to human and animal health [33]. The ER is the largest membrane system in eukaryotic cells, and the expression of ER localization proteins is essential for ER remodeling to form toxisomes [9,40]. The EMC plays a critical role in maintaining lipid homeostasis, signaling, and the biogenesis of many essential proteins in eukaryotic cells [18,32,40,41,42]. However, the role of EMC in *F. graminearum* remains poorly understood. In our study, we identified six EMC subunits (FgEmc1-6) in *F. graminearum* and discovered that EMC is not only crucial for growth and reproduction but also plays significant roles in DON biosynthesis and virulence of *F. graminearum*.

The EMC was first identified in *S. cerevisiae* as an intact membrane protein complex through phenotypic interaction analysis. Its six protein components, EMC1-6, can be co-precipitated to form a hetero-oligomer [43]. Additionally, the membrane proteins SOP4 and YDR056C were co-purified with this complex and subsequently re-identified as EMC7 and EMC10, respectively [15]. In mammals, alongside the aforementioned eight proteins, EMC8 and EMC9 were identified through the mass spectrometry-based endoplasmic reticulum-associated degradation (ERAD) interaction network map [44]. In our study, sequence alignment reveals that the EMC of *F. graminearum* comprises six subunits (FgEmc1-6) (Figure S1). Functional domain analysis revealed that most EMCs contain TMD structural domains, which is consistent with the established role of EMC family proteins [29,30]. Phylogenetic analysis indicates that FgEmc1-6 is highly conserved across eukaryotes (Figure 1). Furthermore, the EMC of *F. graminearum* and *F. oxysporum*, along with those found in other filamentous fungi, exhibit a relatively close phylogenetic relationship, likely due to their similar lifestyles and physiological requirements. In contrast, the EMC of *F. graminearum* shows a more distant phylogenetic relationship with the EMC found in *S. cerevisiae*, *H. sapiens*, and *A. thaliana*, suggesting significant genetic divergence and variation throughout evolutionary history.

The EMC is localized on the ER membrane and plays a crucial role in the folding and assembly processes of membrane proteins. In filamentous fungi, membrane proteins are essential for the uptake and transport of nutrients [45]. The processing of these membrane proteins may be influenced by the EMC, potentially leading to decreased efficiency in nutrient absorption and, consequently, restricting the growth of filamentous fungi [41]. Furthermore, the EMC may also impact hyphal growth through mechanisms such as the regulation of signal transduction, interactions and cooperation with other organelles, and cell cycle regulation [31]. In this study, we investigated the effect of the EMC on the hyphal growth of *F. graminearum*. Our results indicated that, compared to the PH-1 strain, the growth rate of Δ*Fgemc1-6* was lower under various nutritional conditions (Figure 2A,B), and the height of aerial hyphae was also reduced (Figure 2C). This finding is consistent with studies on regulating hyphal vegetative growth and aerial hyphal height by the EMC in *S. cerevisiae* [41,43]. However, it contrasts with observations in *M. oryzae*, where the vegetative growth of Δ*Moemc2* and Δ*Moemc5* was unaffected [32]. This demonstrates that the EMC plays a critical role in the vegetative growth of *F. graminearum*.

The EMC plays a significant role in the development of reproductive structures. Previous studies have demonstrated that EMC is of crucial importance for male reproduction in *Drosophila* [46]. Moreover, the deletion of *EMC10* in mammals results in complete sterility [47,48,49,50]. The results of our study indicate that the deletion of *FgEMC1-6* in *F. graminearum* affects the development of conidia, reduces the number of perithecia, and hinders the maturation and ejection of ascospores (Figure 4), suggesting that FgEmc1-6 have an impact on both the asexual and sexual reproduction of *F. graminearum*.

The EMC was initially identified due to its involvement in the exacerbation of the ER stress response. In *S. cerevisiae*, the deletion of EMC can trigger this stress response [43]. Our study reveals that the Δ*Fgemc1-5* strain exhibits increased sensitivity to dithiothreitol (DTT), while the Δ*Fgemc6* strain shows decreased sensitivity (Figure 3). This difference may be attributed to the deletion or functional alteration of the FgEmc1-5 subunits, which impairs the cells’ ability to effectively process and repair proteins damaged by DTT, leading to the accumulation of misfolded proteins within the ER. Conversely, FgEmc6 may function as a negative regulator in the recognition or repair of misfolded proteins induced by DTT.

The EMC is of essential significance in the adaptive responses of filamentous fungi towards a multiplicity of environmental stressors. For example, in *M. oryzae*, the Δ*Moemc2* and Δ*Moemc5* mutants manifested enhanced susceptibility to CR [32]. Our research findings disclosed that the deletion of *FgEMC1-6* resulted in heightened sensitivity to CR while simultaneously decreasing sensitivity to sorbitol. Remarkably, in response to KCl, only Δ*Fgemc2-4* and Δ*Fgemc6* exhibited elevated sensitivity, whereas Δ*Fgemc1* and Δ*Fgemc5* remained unaltered in their responsiveness to KCl. In the case of H_2_O_2_, only the Δ*Fgemc5* mutant displayed a decreased sensitivity (Figure 3). Collectively, these findings underscore the significance of the EMC in mediating *F. graminearum*’s response to environmental stress. The varied responses among different subunits suggest that the EMC functions as an integrated entity, operating in a coordinated manner, with potential interactions and functional complementarity among its subunits. Disruption of key subunits may compromise the integrity of the EMC, leading to complex responses to diverse stress factors.

In *M. oryzae*, the Δ*Moemc2* and Δ*Moemc5* mutants disrupt appressorium formation, thereby diminishing the virulence during host infection. In *F. graminearum*, DON is pivotal to its pathogenic mechanism and serves as a key determinant of its pathogenicity [8]. The toxisome, a specialized structure, is intricately linked to the biosynthesis of DON and functions as an essential site for its production [11]. Among the various factors involved, the *TRI* gene plays a crucial role in regulating the biosynthesis of DON and significantly influences its production quantity [9]. Moreover, lipid droplets have specialized roles within *F. graminearum*, potentially participating indirectly in DON biosynthesis by supplying the necessary materials or energy [51]. These components are interconnected and interact during the pathogenic process of *F. graminearum*, collectively forming a complex pathogenic network system. Our research indicates that the knockout of EMC affects the formation of toxisomes (Figure 7), the expression of *TRI* genes (Figure 6B), and the generation of LDs (Figure S4), as well as the biosynthesis of secondary metabolite pigments (Figure 6C,D), ultimately leading to a reduction in DON levels (Figure 6A) and a subsequent decrease in the pathogen’s virulence (Figure 5). This study provides significant research targets and directions for further exploration of the pathogenic mechanisms of *F. graminearum*.

## 5. Conclusions

This study identified six EMC subunits in *F. graminearum*. The knockout of each subunit resulted in various phenotypic changes in the fungus, including retarded growth, disrupted reproduction (both asexual and sexual), altered infection structures, and variable responses to environmental stress. Notably, the deletion of EMC significantly reduced deoxynivalenol (DON) biosynthesis and virulence. This reduction is likely attributed to decreased expression of the *TRI* gene, which is crucial for DON synthesis, thereby disrupting toxisome formation and lipid droplet generation and consequently disturbing the molecular processes related to the virulence and fitness of *F. graminearum*.

## Data Availability

Data are available by contacting the corresponding author.

## Supplementary Materials

The following supporting information can be downloaded at https://www.mdpi.com/article/10.3390/jof11020108/s1. Figure S1: Schematic overview of the EMC in *F. graminearum*; Figure S2: The construction and characterization of Δ*Fgemc1-6*. Figure S3: The EMC plays a crucial role in the infection structure formation of *F. graminearum*. Figure S4: The EMC plays an important role in the lipid drop biogenesis of *F. graminearum*. Table S1: Primers used in this study.
