# Supplementary material for: The Endoplasmic Reticulum Membrane Protein Complex Is Important for Deoxynivalenol Production and the Virulence of Fusarium graminearum
Source: J Fungi (Basel). 2025 Jan 31;11(2):108. doi: 10.3390/jof11020108 (PMC11856742; doi:10.3390/jof11020108)
Supplement: Supplementary file 1 [file jof-11-00108-s001.zip › Supplementary Figure Legends.pdf]

Supplementary Figure Legends

Figure S1. Schematic overview of the EMC in *F. graminearum*. A comprehensive bioinformatic analysis was carried out to dissect the domains within the EMC of *F. graminearum*. Notably, one or two transmembrane domains (TMDs) are forecasted to exist in FgEmc1, FgEmc3, FgEmc4, FgEmc5, and FgEmc6. FgEmc1 is projected to harbor pyrroloquinoline quinone (PQQ)-like repeats, which might serve as a crucial scaffold for protein-protein interactions. FgEmc2 is hypothesized to possess tandem tetratricopeptide repeat (TPR) motifs, potentially implicating its role in protein-protein interactions. FgEmc5 is predicted to feature a membrane magnesium transporter (MMgT) domain, suggesting its involvement in magnesium transport processes. Additionally, FgEmc1, FgEmc3, and FgEmc4 are anticipated to have uncharacterized function domains (DUF1620, DUF106, and DUF1077), whose exact roles remain to be elucidated and could potentially hold the key to understanding the unique functions and regulatory mechanisms of the EMC in *F. graminearum*.

Figure S2. The construction and characterization of △*Fgemc1-6*. (A) The schematic strategy for the deletion of *FgEMC1-6* in *F. graminearum* is depicted. The *FgEMC* entire coding regions were replaced with the hygromycin-phosphotransferase (*HPH*) gene by means of homologous recombination. (B) Amplification of target gene fragments and diagnostic PCR were used to identify the positive transformants.

Figure S3. The EMC plays a crucial role in the infection structure formation of *F. graminearum*. The infection cushions generated by PH-1 and *ΔFgemc1-6* on the wheat lemma at 3 days post-inoculation (dpi) were inspected using scanning electron microscopy (SEM) at magnifications of ×800 and ×2000.

The representative micrographs presented defect in infection cushion formation in *ΔFgemc1-6*. Abbreviations: FS Foot structures, IC infection cushion, IH infection hypha, LA lobate appressorium, RH runner hyphae.

Figure S4. The EMC plays an important role in the lipid drop biogenesis of *F. graminearum*. PH-1 and *ΔFgemc1-6* were grown in CM and TBI medium respectively. The distribution of lipid droplets in vivo was detected via fluorescence using Nile Red staining of vegetative hyphae. Scale bars, 10 μm.
