# Supplementary material for: The Endoplasmic Reticulum Membrane Protein Complex Is Important for Deoxynivalenol Production and the Virulence of Fusarium graminearum
Source: J Fungi (Basel). 2025 Jan 31;11(2):108. doi: 10.3390/jof11020108 (PMC11856742; doi:10.3390/jof11020108)
Supplement: Supplementary file 1 [file jof-11-00108-s001.zip › Table S1. Primers used in this study.pdf]

Supplementary Information

The ER membrane protein complex (EMC) is important for DON production and virulence of *Fusarium graminearum*

Table S1. Primers used in this study

Primer name	Oligonucleotide sequence (5'-3')	Application
EMC1-M-F	CAATGGCGGTGGTTTCTTGC	
EMC1-M-R	GACGCTTCCAGTCGACCTCG	
EMC1-K1-F	GGGCCAATTAGCTCTAATGATTTCATG	
EMC1-K2-R	GCACAAGTCGGAACCCAAGC	
EMC2-M-F	CAACCCCAAGGCTTTCTCAGC	
EMC2-M-R	GCCTGGTTCAACTTCTTCAGGG	
EMC2-K1-F	CGCCAATCTCCAAGAGAACCTTG	
EMC2-K2-R	CAACAGCGAGCAGTTTGCAGAC	
EMC3-M-F	GAAAAGACCATGCGCGAGCAG	
EMC3-M-R	CGTTCAAGTTCTCGACCTCAGC	
EMC3-K1-F	CGCCCTGCTCTCTAAGCATTG	
EMC3-K2-R	GTGACGAACTTCCCTGCACAC	For identification of *FgEMC1*，*FgEMC2*，*FgEMC3*，*FgEMC4*，*FgEMC5* and *FgEMC6* deletion transformants
EMC4-M-F	CACCAACTCCAAGGTCAGCAC	
EMC4-M-R	CCTTACGGTAATAAGCCCATGGC	
EMC4-K1-F	CCCAATAATGTCGGGGAACAAGG	
EMC4-K2-R	GATTTTGGTGACATGGCAGGACG	
EMC5-M-F	CTTCGAGATCACCCAGCTTC	
EMC5-M-R	CGCTAGACGACGCCATCATG	
EMC5-K1-F	CTCGAAACCGAAAAAGGCCAAAC	
EMC5-K2-R	CTTGGGAGTTCAAACCCACGAG	
EMC6-M-F	CCAGATCAACCCAATCGTTCCTG	
EMC6-M-R	CCATGTCAAGACGAAACCCGAG	
EMC6-K1-F	GCGATCTGCTTGAGGTAAGGC	

EMC6-K2-R	GCCATCTACACCAACCGCAC	
H1-F	GGCTTGGCTGGAGCTAGTGGAGGTCAA	For amplify hygromycin H1 fragmen
H1-R	GTATTGACCGATTCCTTGCGGTCC	
H2-F	GATGTAGGAGGGCGTGGATATGTCCT	For amplify hygromycin H2 fragmen
H2-R	GTATTGACCGATTCCTTGCGGTCCGAA	
EMC1-A-F	CGTAGCAAGGGACTCTTGAATCATC	
EMC1-A-R	GATGTGTTGACCTCCACTAGCTCCAGCCA AGCCTGTGGTTGTGTTGAGGGCGTTTATG	For amplify *EMC1*A fragmen
EMC1-B-F	AAGGAATAGAGTAGATGCCGACCGCGGGT TCTGAAAAGTGAGGGCATGTCATATTAGG	For amplify *EMC1*B fragmen
EMC1-B-R	GTCTTGGGAGGGCAAACTCC	
EMC2-A-F	GCGGGGTCCTGACTAAATTTTCC	
EMC2-A-R	CATTGATGTGTTGACCTCCACTAGCTCCAG CCAAGCCGTCGCTATGGGGTGAATCCGG	For amplify *EMC2*A fragmen
EMC2-B-F	CAAAGGAATAGAGTAGATGCCGACCGCGG GTTTCGATCATGTACGCAAGGGTTTAGAC	For amplify *EMC2*B fragmen
EMC2-B-R	GAATGTCGGTCAAGCAGCCATG	
EMC3-A-F	GGAAACAGTAACGCGCTTGGG	
EMC3-A-R	CCTCCACTAGCTCCAGCCAAGTCGATATTC CAAGAGGTGCAATTGAGTATATGGGAAT	For amplify *EMC3*A fragmen
EMC3-B-F	AAAGGAATAGAGTAGATGCCGACCGCGGG TTCATAGAACGAGCAAACGTATATACGCC	For amplify *EMC3*B fragmen
EMC3-B-R	CGTTGTGGCCTCACTGATTCC	
EMC4-A-F	CGGGCCATCATAAATCGACTTGG	
EMC4-A-R	TGTTGACCTCCACTAGCTCCAGCCAAGCC GTTGTATCACTTCGGAGTCCGTATATTCG	For amplify *EMC4*A fragmen
EMC4-B-F	CAAAGGAATAGAGTAGATGCCGACCGCGG GTTCACTTTTATGTTGCCCTCAATGCTTC	For amplify *EMC4*B fragmen
EMC4-B-R	CTGTTGTGGCCGACATCTCATC	

EMC5-A-F	CTCTATGGGCGATCCATCGAC	
EMC5-A-R	GACCTCCACTAGCTCCAGCCAAGCCTGGA<br>ATAGATACACTGAAGACAAGTGTTGGCTAG	For amplify *EMC5*A fragmen
EMC5-B-F	AATAGAGTAGATGCCGACCGCGGGTTCTTT<br>GACTGCTCATGGAAATATGTATCGG	For amplify *EMC5*B fragmen
EMC5-B-R	CTAAGAAAGTCATGCCAAGTCACTTGC	
EMC6-A-F	GAATCCAATCAAGGGCACCTGAC	
EMC6-A-R	GATGTGTTGACCTCCACTAGCTCCAGCCA<br>AGCCCGTTGCGGATATTATAAGATGGCCG	For amplify *EMC6*A fragmen
EMC6-B-F	AAGGAATAGAGTAGATGCCGACCGCGGGT<br>TGTCAACAGTACCAGGAGTCTACATCTAC	For amplify *EMC6*B fragmen
EMC6-B-R	GTTGTTGGGCCTTTTCCACCC	
GAPDH-F	CTTACTGCCTCCACCAACTG	
GAPDH-R	TGACGTTGGAAGGAGCGAAG	
TRI1-QF	TTGAACACTACCTCGGTGCT	
TRI1-QR	AGTTCGCGAGCATTCTTGAC	
TRI4-QF	CCTGGTCTGGTCACCATTCT	For *TRI* gene qRT-PCR analysis
TRI4-QR	ATGGCCAGTGTCCTTGAAGT	
TRI5-QF	GAGTGTTTCATGCATGGCTACGTC	
TRI5-QR	CTGAGCCTCCTTCACATCGTCC	
TRI6-QF	CTGAGGGCATTCTGAGTAGCGACA	
TRI6-QR	CGTTATGTTTATCGGCACTTTG	
TRI10-QF	GCGACAGGAGCAAGAACATAA	
TRI10-QR	GGCGGCGTAAATCTGAGTG	
GIP1-QF	TGCGGTATCAGGTCACAAA	
GIP1-QR	ATCAAAGTCTCCCACCGTGAA	
GIP2-QF	CACCAGCCCTACACCATCTAA	
GIP2-QR	TTTCCAAAGCGAGAAACAGC	
PKS12-QF	TGGTGTAGATGCTGTTCGTGT	

PKS12-QR	TGAACTTTTCGAGGACGGAT	For qRT-PCR analysis of pigment gene
AURJ-QF	AAAAAGCAGCCAAGGAGCAT	
AURJ-QR	TTCTGATGACACGCTCCCGTA	
AURF-QF	ATCTTCAGTCTTGACCATCCC	
AURF-QR	TACCCAAGATGTTCTGGCAA	
FgEMC1-mCherry-F	GTTCTCATCACCATCACCATCACTCGAGTAAGGCTCTCGGGGGTAGGTTC	For FgEMC1 subcellular localization
FgEMC1-mCherry-R	CCTCCTCGCCCTTGCTCAATAGAGAAAATACAGTACTTTATTTCCATTTACAGCATC	
FgEMC2-mCherry-F	GTTCTCATCACCATCACCATCACTCGAGAACCGGACCTGAGCCAGATC	For FgEMC2 subcellular localization
FgEMC2-mCherry-R	CATGTTATCCTCCTCGCCCTTGCTCAATCACCGGACAACCTCTGAAGACG	
FgEMC3-mCherry-F	GTTCTCATCACCATCACCATCACTCGAGGCATTCGGTCTTGTACCATTGGC	For FgEMC3 subcellular localization
FgEMC3-mCherry-R	GCCATGTTATCCTCCTCGCCCTTGCTCAACTACGATTTGATACCCGCCAATAGAC	
FgEMC4-mCherry-F	GTTCTCATCACCATCACCATCACTCGAGTACAGTAGCCTCGACGAATTCTCTAACATG	For FgEMC4 subcellular localization
FgEMC4-mCherry-R	CATGTTATCCTCCTCGCCCTTGCTCAATTATAGAGCTGCGACAGCATGTTC	
FgEMC5-mCherry-F	CGTGGTTCTCATCACCATCACCATCACTCGAGAAACGTCACCTTCGAGATCACCC	For FgEMC5 subcellular localization
FgEMC5-mCherry-R	GATGGCCATGTTATCCTCCTCGCCCTTGCTCAAGTCACATAATTCTCTGCGTCGCTAG	
FgEMC6-mCherry-F	GTGGTTCTCATCACCATCACCATCACTCGAGGGTCGTGCAGCCAGTCCATTC	For FgEMC6 subcellular localization
FgEMC6-mCherry-R	GTTATCCTCCTCGCCCTTGCTCAACTATGCCCTAACTAAACCATAAAATAATGTCCATG	
