# Supplementary figures and images for: The Endoplasmic Reticulum Membrane Protein Complex Is Important for Deoxynivalenol Production and the Virulence of Fusarium graminearum
Source: J Fungi (Basel). 2025 Jan 31;11(2):108. doi: 10.3390/jof11020108 (PMC11856742; doi:10.3390/jof11020108)

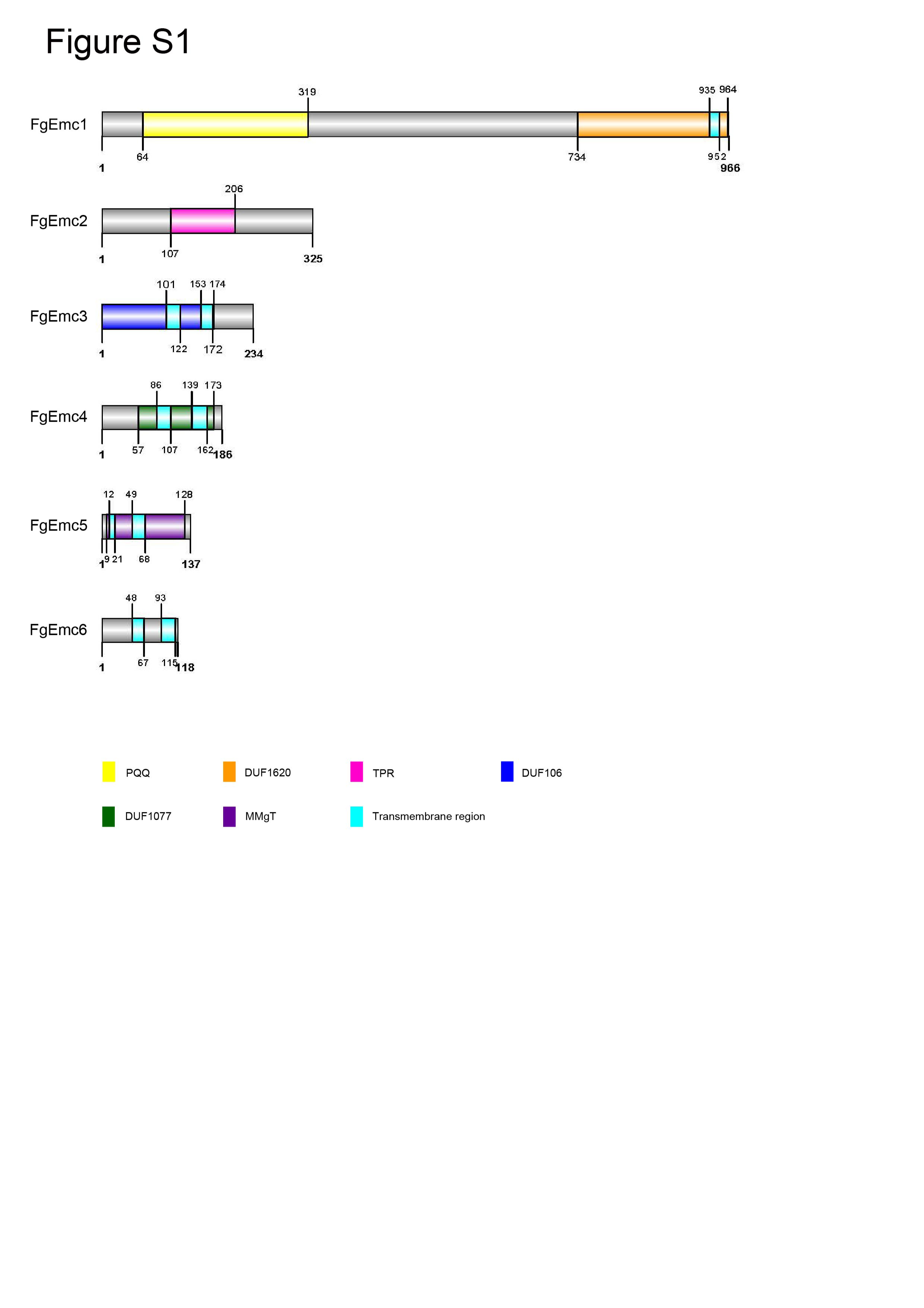

Supplement: Supplementary file 1 [file jof-11-00108-s001.zip › Figure S1.tif]

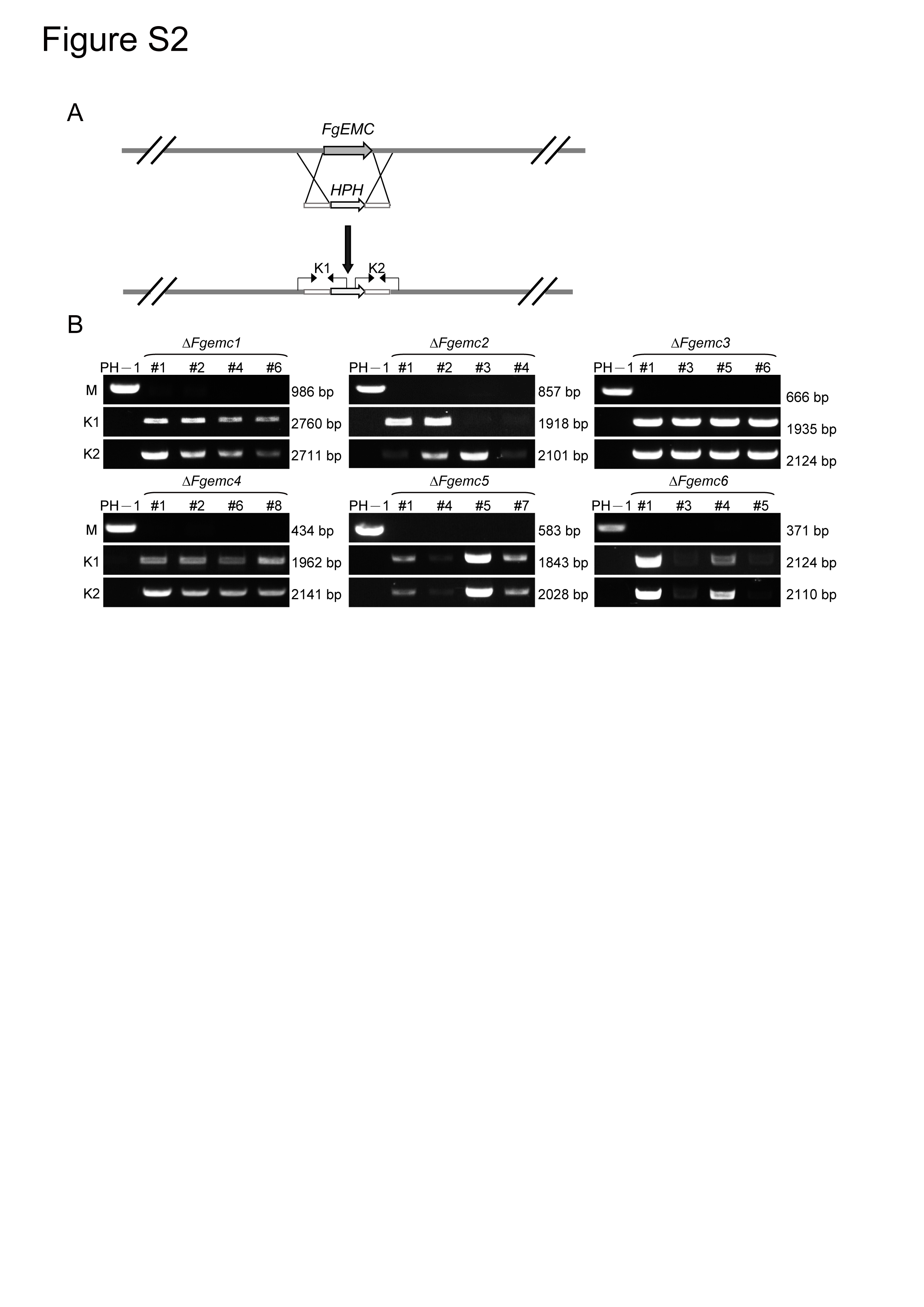

Supplement: Supplementary file 1 [file jof-11-00108-s001.zip › Figure S2.tif]

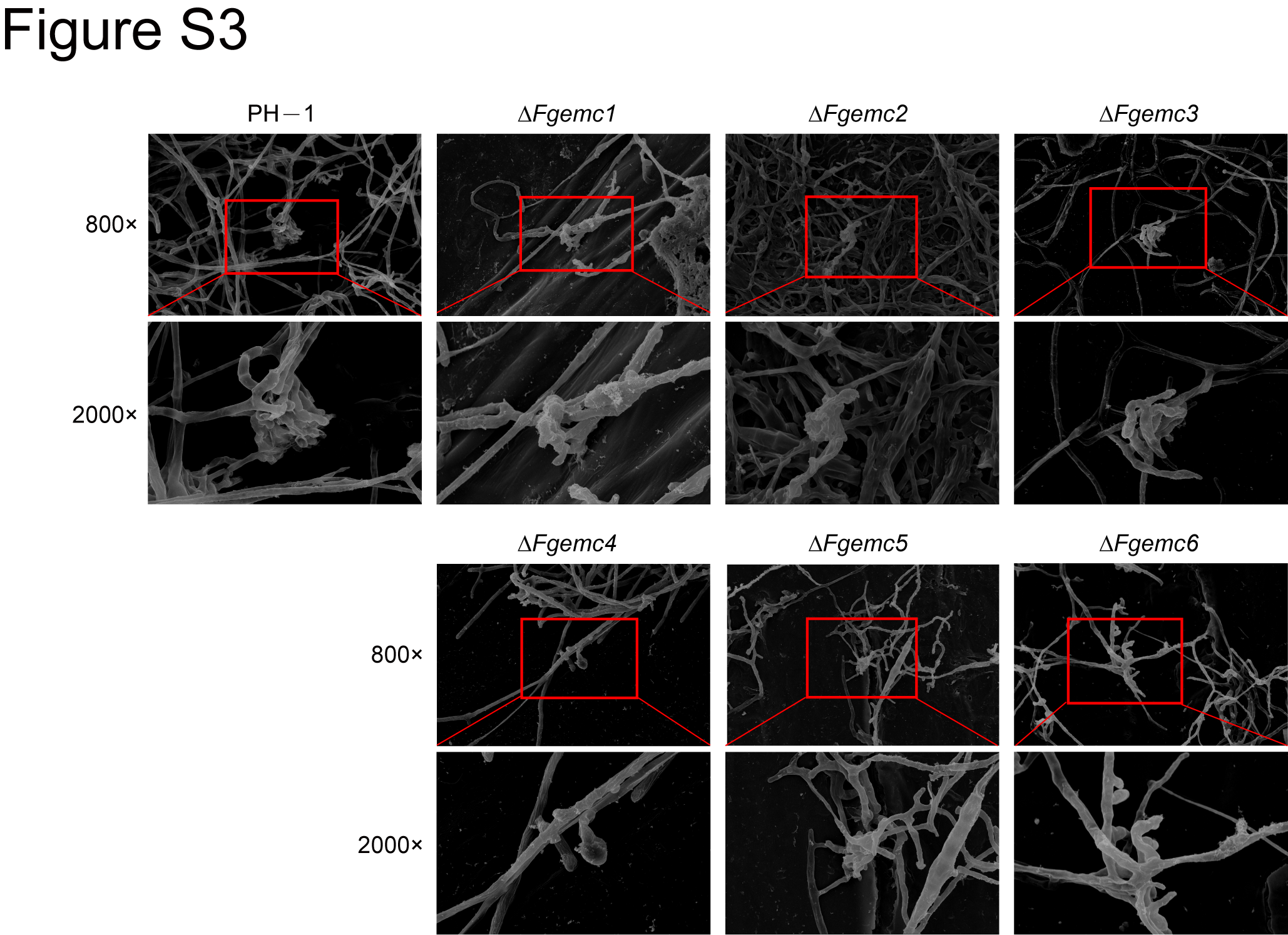

Supplement: Supplementary file 1 [file jof-11-00108-s001.zip › Figure S3.tif]

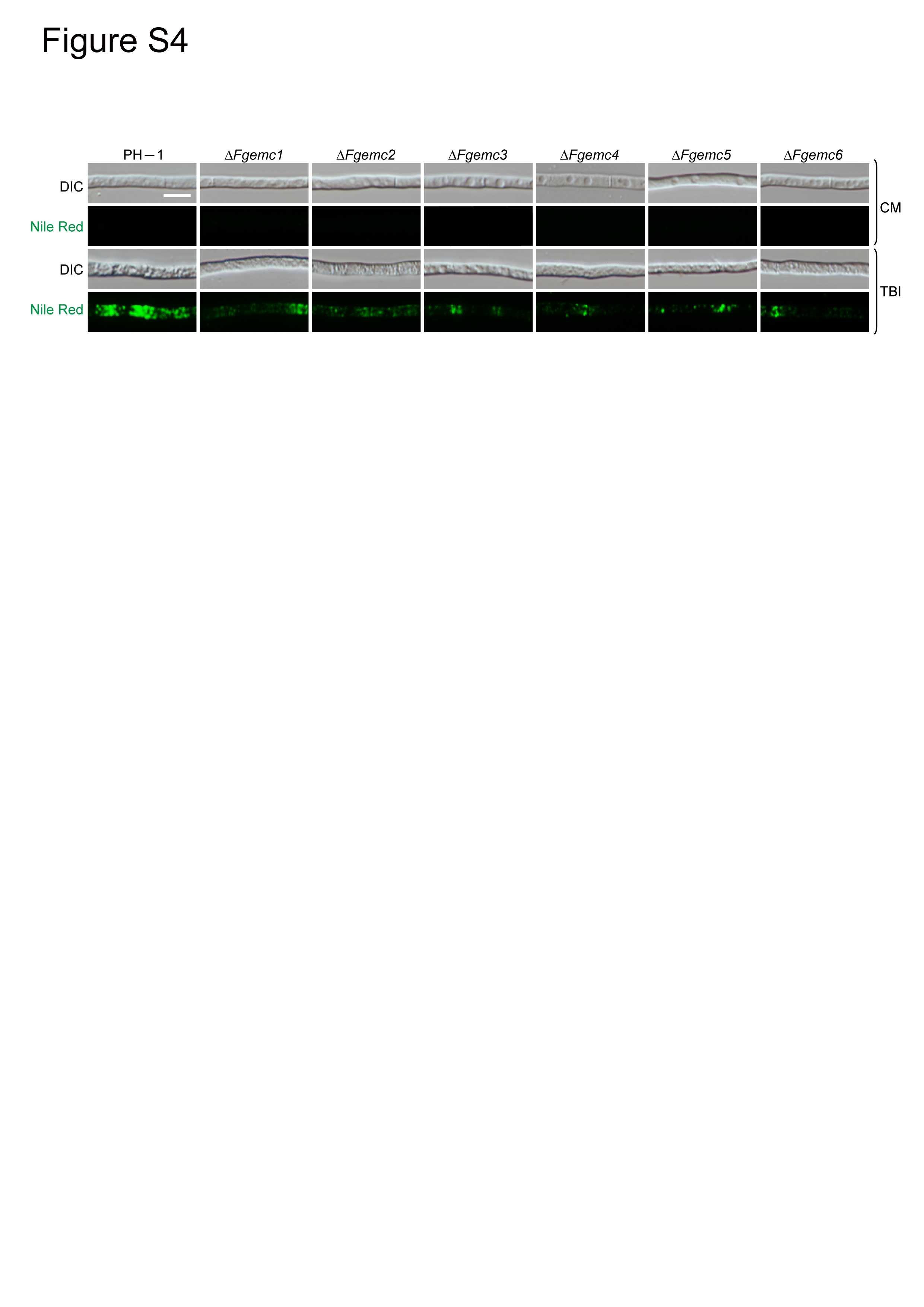

Supplement: Supplementary file 1 [file jof-11-00108-s001.zip › Figure S4.tif]
